# Quality of life in women undergoing urinary diversion for bladder cancer: results of a multicenter study among long-term disease-free survivors

**DOI:** 10.1186/1477-7525-11-43

**Published:** 2013-03-12

**Authors:** Mauro Gacci, Omar Saleh, Tommaso Cai, John L Gore, Carolina D’Elia, Andrea Minervini, Lorenzo Masieri, Claudia Giannessi, Michele Lanciotti, Virginia Varca, Alchiede Simonato, Sergio Serni, Giorgio Carmignani, Marco Carini

**Affiliations:** 1 Urologic Clinic I, University of Florence, AOU Careggi, Florence, Italy; 2 Urologic department, University of Genova, Genova, Italy; 3 Department of Urology, Santa Chiara Hospital, Trento, Italy; 4 Department of Urology, University of Washington, Fred Hutchinson Cancer Research Center Seattle, Seattle, WA, USA; 5 Department of Urology, University of Florence, Florence, Italy

**Keywords:** Urinary diversion, Orthotopic urinary reservoirs, Bladder cancer, Quality of life

## Abstract

**Purpose:**

Women undergoing radical cystectomy (RC) and urinary diversion for bladder cancer experience substantial limitations in health-related quality of life (HRQOL). However, the level of discomfort caused by different urinary diversion has been never evaluated in long term survivors. The aim of this multicenter study is to evaluate differences in HRQOL among recurrence-free women undergoing cutaneous ureterostomy (CUS), Bricker's ileal conduit (BK-IC) and Orthotopic neobladder VIP (ONB-VIP) in disease-free females treated with radical cystectomy (RC), with long-term follow up (mean 60.1 months; range 36-122 months).

**Materials and methods:**

All consecutively treated female patients from two urological institutions who underwent RC and urinary diversion from January 2000 to December 2008, with no evidence of tumor recurrence at a minimum follow up of 36 months, were included. Patients received the European Organisation for Research and Treatment of Cancer (EORTC) generic (QLQ-C30) and bladder cancer-specific instruments (QLQ-BLM30) and the Functional Assessment of Cancer Therapy for Bladder Cancer (FACT-BL). Clinical data and questionnaire results were analyzed in order to evaluate the HRQOL differences among diversion groups.

**Results:**

We identified 37 females (median age: 68, range 45–82 years), including 12 status-post CUS, 16 who underwent BK-IC, and 9 who underwent ONB-VIP. Most were healthy (24/37 with no comorbidities, 4/37 Charlson 1-2, 9/37 Charlson 3 or greater – we didn’t considered bladder cancer in Charlson evaluation because bladder cancer was the main inclusion criteria). Women undergoing CUS endorsed worse FACT-BL scores compared with BK-IC and ONB-VIP patients, worse HRQOL regarding physical and emotional well-being (p=0.008 and p=0.02, respectively), and a trend toward worse EORTC QLQ-C30 scores for appetite loss and fatigue (p=0.05 for both).

**Conclusions:**

In our study long-term disease-free females treated with CUS endorsed worse HRQOL compared with women who underwent BK-IC or ONB-VIP, mostly due to worse physical and emotional perception of their body image.

## Introduction

Bladder cancer (BC) ranks ninth in worldwide cancer incidence. It is the 7th most common cancer in men and the 17th most common cancer in women with more than 330,000 new cases each year and more than 130,000 deaths per year [1]. According to the European Association of Urology (EAU) and National Comprehensive Cancer Network guidelines (NCCN), radical cystectomy (RC) is the standard treatment for localized muscle-invasive bladder cancer and high-risk non-muscle-invasive cancers resistant to endovesical chemotherapy [2].

In contemporary series, patients may achieve excellent long-term functional and oncologic outcomes when treated at high-volume institutions by experienced surgeons. Beyond the primary aim of disease control, removal of the bladder requires reconstruction of a lower urinary tract. Surgeons can reconstruct complex urinary diversions that also safeguard patients’ health-related quality of life (HRQOL) [3], but this is a highly morbid surgical procedure associated with significant changes in urinary and sexual function, strained relationships, and psychosocial stress [4]. For this reason, in the last years, the evaluation of HRQOL after such an impacting procedure has garnered increasing interest [5].

Several studies have attempted to evaluate HRQOL following RC and focused on the effects of various types of lower urinary tract reconstruction [6,7]. Hobisch et al. found that HRQOL was preserved to a higher degree with a continent orthotopic neobladder (ONB-VIP: where last tract of ileal bowel is reconfigured into a sphere and sewn to the urethral stump to facilitate voiding per urethra) compared with an incontinent diversion like cutaneous ureterostomy (CUS: where ureters are connected to the surface of the abdomen with the formation of an opening stoma) and ileal conduit urinary diversion (BK-IC: where the urine is diverted to a bag on the skin, made by short tract of small bowel) [8]. Similarly, Heningssohn et al., compared a cohort of patients that underwent RC and orthotopic bladder substitution with a matched non-operative control group and demonstrated preserved global quality of life [9]. Yet, other studies comparing different types of urinary diversion were unable to confirm superiority of one type of reconstruction with regard to HRQOL [10-12]. Recently, Hedgepeth, et al., showed no difference in body image scores between IC and ONB patients after surgery [13]. However, many of these reports have no long-term follow up, and often include very few recurrence-free female patients.

The aim of this study is to evaluate differences in HRQOL between different forms of urinary diversion in a consecutive series of female who had RC for muscle-invasive bladder cancer. We concentrated on long term (3years or more) recurrence-free females in order to assess HRQOL in a cohort that would not be biased by bladder cancer treatments.

## Materials and methods

### Study design

In order to evaluate the differences in terms of HRQOL among women treated with CUS, or BK-IC or ONB-VIP after RC for clinically localized bladder cancer, all consecutively female patients treated in two urological institutions from January 2000 to December 2008 without any evidence of tumor recurrence after at least 36 months from surgery were considered for this study. We enrolled disease-free patients in order to exclude bother related to adjuvant and salvage therapies and avert the social/emotional implication of disease progression. Another reason to enroll patients after at least 36 months after surgery was to avoid the biases of peri-operative care (more complex urinary diversions require more intensive nursing care in the postoperative recovery period).

All patients were retrospectively assessed with the following self-administered questionnaires (they had help to filling the questionnaire in if it was needed): the European Organization for Research and Treatment of Cancer (EORTC) generic (QLQ-C30) and bladder cancer-specific surveys (QLQ-BLM30) and the Functional Assessment of Cancer Therapy for Bladder Cancer (FACT-BL). For analysis, patients were stratified according to urinary diversion type.

Subsequently the responses were entered into a database. Data from the questionnaires were analyzed in order to test differences among the groups. Informed consent was obtained from all patients. The study was conducted in accordance with the principles of research involving human subjects as expressed in the Declaration of Helsinki and with Good Clinical Practice.

### Inclusion and exclusion criteria

Included patients were at least 18 years of age, who underwent RC and urinary diversion for clinically localized bladder cancer in accordance with EAU criteria [14]. Only disease free females at least 36 months after RC were included in this study. All enrolled patients were able to ply the study design, including to self compile the selected questionnaires. We excluded patients with major concomitant medical or psychological diseases, including those with remarkable bowel disease and those with previous lower tract surgery, with the exception of staging TURBT. Likewise, patients previously treated with neoadjuvant chemotherapy or radiation therapy were excluded.

### Surgical techniques and surveillance

Patients were followed according to the surveillance schedules suggested in EAU guidelines [14]. RC with pelvic lymphadenectomy was performed as described by Skinner, et al [15]. ONB-VIP was performed in patients without locally advanced disease (including absence of hydroureteronephrosis) or bladder neck involvement, normal renal and bowel function, and functional capability to manage urinary diversion. For those women who did not meet criteria for ONB, we performed a non-orthotopic urinary diversion. All procedures were performed by two dedicated surgeons.

We classified perioperative complications according to the Clavien–Dindo classification [16]. For risk adjustment, we calculated each patient’s Charlson Comorbidity Index [17]. Follow-up after urinary diversion was scheduled every 3 months during the first 2 years and subsequently every 6 months for 5 years or more. Follow up included general physical examination, routine serum chemistries, urinary cytology, total abdominal US, chest x-ray, CT urography, and cystoscopy (only in very selected cases).

### HRQOL measurement and questionnaire descriptions

We used the general and bladder cancer-specific HRQOL questionnaires EORTC QLQ-C30 [18], EORTC QLQ-BLM30 [19] and FACT-BL [20]. All questionnaires were self-administered during a scheduled follow-up visit.

#### EORTC QLQ-C30

We used the Italian version of the EORTC QLQ-C30. In brief, the EORTC QLQ-C30 is a 30-item questionnaire composed of multi-item scales and single items that reflect the multidimensionality of the quality-of-life construct [18]. It incorporates five functional scales (physical, role, cognitive, emotional, and social), three symptom scales (fatigue, pain, and nausea/vomiting), and a global health and quality-of-life scale. The remaining single items assess additional symptoms commonly reported by cancer patients (dyspnea, loss of appetite, sleep disturbance, constipation, and diarrhea), as well as the perceived financial impact of cancer and its treatment [18]. The majority of questions were assigned a score from one to four (1=not at all, 4= very much) [18]. Questions 29 and 30 were assigned a score from 1 to 7. All scores were linearly transformed to a 0-100 scale. Lower score matches to higher HRQOL

#### EORTC QLQ -BLM30

We used the validated Italian version of QLQ-BLM30 from EORTC to assess bladder cancer-specific HRQOL [19]. All questions are specific to patients treated for muscle-invasive bladder cancer. The EORTC QLQ-BLM30 was developed to evaluate the HRQOL detriments associated with having a urostomy and measures the effects of the urostomy appliance on patients’ HRQOL following urinary diversion [19]. Patients with continent cutaneous pouches can answer questions concerning their ability to perform catheterization. Due to the fact that cystectomy and urinary diversion involves major physical changes, the instrument is also designed to assess any changes in the patient’s body image. Moreover, it evaluates the physical changes that affect the patient’s sexual life and the patient’s mood [18]. Finally, the bowel aspect was evaluated in relation to presence of intestinal reconfiguration. Each question was assigned a score from one to four (1=not at all, 4= very much) [19]. All scores were linearly transformed to a 0-100 scale. Lower score matches to higher HRQOL

#### FACT (BL-VCI)

The FACT-BL similarly evaluated patients’ bladder cancer-specific HRQOL. We used the validated Italian version of FACT-BL [20]. FACT-BL is useful for comparisons of the HRQOL of patients with various diversions including an ileal conduit, a continent cutaneous reservoir, or an orthotopic neobladder. The scores were calculated from the FACT-BL questionnaire and subsetted into the domains of: physical well-being (PWB), social/family well-being (SWB), emotional well-being (EWB), functional well-being (FWB), total FACT-G score (which is incorporated into the FACT-BL), overall bladder cancer-specific subscale, and total FACT-BL score (FACT-G + bladder cancer subscale) [20]. The survey is composed of 39 questions. Each question was assigned a score from one to four (1=not at all, 4= very much) [20]. A total of 17 additional questions were added to the FACT-G to create the Vanderbilt Cystectomy Index (FACT-VCI) [20]. Lower score matches to higher HRQOL.

### Statistical analysis

As null hypothesis we assumed that there was no difference among the urinary diversion groups in terms of HRQOL. Continuous, normally distributed variables were reported as the mean value with standard deviation (SD). Summary scores and responses to individual items are presented using descriptive statistics.

All data were stratified according to urinary diversion with univariate ANOVA analyses (all diversions) and with unpaired sample T tests. Statistical significance was achieved if p was <0.05. All reported p-values were two-sides. All data were recorded, collected and analyzed by using Statistical Package for the Social Sciences 16.0 for Microsoft (SPSS, Inc., Chicago, Illinois, USA).

## Results

We enrolled 41 female patients who had RC for muscle invasive bladder cancer without any evidence of tumor recurrence after at least 36 months from surgery. 4 patients were excluded: 1 had serious inflammatory bowel disease and 3 had previous lower tract genitourinary surgery. Overall, a total of 37 females patients were included in the study. Mean age at surgery was 67.3 ± 8.7 years; mean age at follow-up was 73.1 ± 8.7 years. Clinical and pathological characteristic of the patients at surgery, stratified by urinary diversion type, are described in Table 1.

**Table 1:** Clinical and pathological characteristics of our study patients, stratified according to different urinary diversion

		**CUS**	**Bricker**	**VIP**
		12	16	9
**Age at follow up, mean ± SD**		75.3 ± 10.8	74.4 ± 8.8	71.8 ± 7.0
**Pathological stage classification**	pT1 /Tis	4 (%)	6 (%)	0 (%)
	pT2	3 (%)	7 (%)	4 (%)
	pT3	5 (%)	3 (%)	5 (%)

Twelve women were treated with CUS, 16 with BK-IC and 9 with ONB-VIP. Comorbidity counts were 0 in 24 patients, 1-2 in 4 patients, and 3 in 9 patients. All groups were similar according to clinical, pathological and peri-operative characteristics at the time of RC. All patients were disease-free at most recent follow-up (mean 60.1 months; range 36-122 months).

### Functional outcomes

All included patients completed all the questionnaires. More remarkable subscores of all questionnaires are reported in Table 2. The EORTC QLQ-C30 identified a trend toward worse HRQOL for “appetite loss” and “fatigue” among CUS patients compared with BK-IC or ONB-VIP (1.50 vs 1.12 and 1.96 vs 1.51, respectively, p=0.05 for both analyses). The FACT-BL questionnaire demonstrated significantly worse “physical well-being” (Additional file 1: Figure S1) and “emotional well-being” (Additional file 2: Figure S2) for CUS compared with BK-IC or ONB-VIP (p=0.008 and p=0.02, respectively). No other differences in questionnaire results among the three urinary diversions groups was reported. In relation to sexual function, no difference among the diversion groups was recognized.

**Table 2:** Average scores of any considerable specific subscales of questionnaires, stratified according to different urinary diversion

**Questionnaire**	**Subscale**	**CUS av**	**BK av**	**VIP av**	p*
**EORTC QLQ C 30**	Total score	28.1 ± 8.7	21.5 ± 6.2	23 ± 2.2	
	*Physical function*	1.8	1.3	1.4	(p=n.s.)
	*Diarrhea*	2.5	1.0	1.0	(p=n.s.)
	*Appetite loss*	**1.5**	**1.1**	**1.1**	**(p=0.054)**
	*Fatigue*	**2.0**	**1.5**	**1.6**	**(p=0.053)**
**EORTC QLQ BLM 30**	*Total score*	7.3 ± 2.6	6.8 ± 2.0	6.7 ± 2.0	
	*Body image*	2.3	2.0	1.4	(p=n.s.)
	*Sexual functioning*	0.6	0.5	0.3	(p=n.s.)
**FACT-**BL	*Total score*	7.8 ± 2.3	6.2 ± 2.1	7.1 ± 1.4	
	*Social well being*	1.9	2.0	2.1	(p=n.s.)
	*Functional well being*	1.8	2.0	2.0	(p=n.s.)
	*Physical well being*	**1.3**	**0.6**	**0.7**	**(p=0.008)**
	*Emotional well being*	**1.7**	**1.2**	**1.3**	**(p=0.024)**

In bold significant p value (*unpaired T test: CUS vs BK+VIP).

## Discussion

In the last two decades, there has been a growing recognition of the importance of HRQOL among patients undergoing invasive surgical procedures. Therefore, some patients achieve durable cancer-free outcomes. It becomes necessary to safeguard the patients’ HRQOL related to their bladder cancer and its treatment [3].

In our trial we investigated with validated instruments the overall quality of life of a very selected population of patients. In particular, we demonstrated that women undergoing CUS experience a loss of appetite and a worsening of fatigue in daily activities, inducing a worsening of both the physical and emotional well being.

In our paper some aspects are interesting: the study was conducted on long term disease free survivors after RC for clinically localized bladder cancer. This specific population allows us to avoid biases related to the initial postoperative worse HRQOL and/or the fear for tumor recurrence after RC [21]. Moreover, we excluded from our study both adjuvant-related bother and social/emotional implication of disease progression and obtain health-related HRQOL results with minimal biases.

A further strength of this paper is the use of more than one specific validated questionnaire. In the majority of scales and items no differences were found between treatment groups in several clinical trials, such as in our study: therefore, the use of several questionnaires allow us to define the minimal variances across different treatment groups.

The quality of life in patients undergoing cystectomy should take into account many aspects that must be evaluated in order to have data that comes as close as possible to the real situation of the patients themselves. No questionnaire, even if validated, disease specific and well-built, will take into account all the aspects necessary to have a complete evaluation. For this reason we decided to use different and validated questionnaires to evaluate more aspects as possible like: functional, symptoms, global health aspects (*EORTC QLQ-C30),* the effects of the urostomy (*EORTC QLQ -BLM30)* and patients well-being (FACT-BL).

Several Authors, by using a single HRQOL questionnaire, did not find any significant correlation between HRQOL and the urinary diversion type. Using several questionnaires it is possible to evaluate different aspects regarding patients HRQOL. Consequently more differences, if exist, can be measured. Furthermore the differences obtained can be evaluated by analyzing several aspect of patients life that only with multiple items, provided by multiple questionnaires, would be possible. Saika et al., by using the EORTC C-30 questionnaire only, concluded that the type of urinary diversion does not appear to be associated with a different HRQOL [22]. In our study, beyond the differences we obtained in EORTC C-30 regarding “appetite loss” and “fatigue” in favor of BK-IC/ONB-VIP, the FACT-BL questionnaire showed a significantly worse HRQOL for CUS compared with BK-IC/VIP-ONB regarding “physical well being” and “emotional well being”. This data can be explained by the fact that patients with CUS showed worse HRQOL outcomes when compared with BK-IC/VIP-ONB merely for “appetite loss” and “fatigue” (EORTC QLQ -C30).

The suggested link between the worsening of “appetite loss” and “fatigue” (EORTC QLQ -C30) and the decline of “physical well being” and “emotional well being” (FACT-BL) could be investigated only by using different questionnaires.

Moreover, the multiplicity of aspects investigated allows us to evaluate possible differences between what is expected based on surgical and anatomical data in literature and the real quality of life of the patient. For example during cystectomy the neurovascular bundle could be damaged [23] causing the worsening of the sexual female quality of life and sexual desire. This aspect should be affect emotional wellbeing inducing a decline of the last one. Under these conditions by analyzing both the sexual aspect (BLM-30) and emotional wellbeing (FACT-BL) we found that there is no relation between them, in fact we observed a decline physical and emotional wellbeing and no differences in sexual function.

The present study has several limitations that should be considered when interpreting our results: in particular, this is a retrospective, cross-sectional, non randomized study in a limited number of patients. It caused a lack of information about baseline pre-treatment HRQOL evaluation, even if all data have been collected and assumed by the case history. However, the possibility of baseline differences in terms of HRQOL among the groups cannot be ignored. Some items, not specific for urological patients, such as mood disturbance or cognitive status were not included in our evaluation. Because this was not a randomized clinical trial, certain biases may have been introduced into analysis that were not properly controlled for and may have impacted the outcome, including the policies regarding urological operations in two different urologic centers involved in this study. In particular, as reported in the “2nd International Consultation on Bladder Cancer recommendations on the reconstructive options after radical cystectomy”, there are no evidences to recommend one form of diversion over another one, in females [24]. Consequently, the two referral centers that performed RC did not base the choice for urinary diversion on standard criteria, but have tailored the UD case-by-case. Therefore, we can suppose that the loss of appetite and the fatigue as well as the physical and emotional well-being scores in the CUS group may depend not only on urinary diversion but also on patients’ characteristics.

Finally, as suggested by Studer, there is a lack of specific items regarding the management of the stoma [4]. We need further larger, prospective longitudinal and randomized studies, in order to confirm these findings among the urinary diversion groups.

## Conclusions

In conclusion, based on data from a cross-sectional, non randomized study in a limited number of cases, females treated with cutaneous ureterostomy seem to be affected by a worse HRQOL when compared with those treated with Bricker or Paduan Ileal Neobladder, for the deterioration of both the physical and emotional perception of their body image.

Moreover, there is no an exclusive questionnaire that should be used as gold standard to measure HRQOL in female after cystectomy: therefore, several validated questionnaires, investigating different items, scales and scores, have to be used to measure all the aspects of quality of life after RC in female, in future studies.

Further randomized prospective studies, based on the data of our pilot study, are needed to address this important outcome.

## Abbreviations

RC: Radical cystectomy; HRQOL: Healt-related quality of life; CUS: Cutaneousureterostomy; BK-IC: Bricker-Ileal conduit; VIP-ONB: Paduan Ileal Neobladder- Orthotopic neoblader; FACT: Functional assessment cancer therapy; EORTC QLQ-BLM 30: European Organisation for Research and Treatment of Cancer Quality of life Questionnaire bladder muscle invasive; EORTC-QLQ-C30: Treatment of Cancer Quality of life Questionnaire Cancer.

## Competing interest

The author(s) declare that they have no competing interests.

## Authors’ contributions

MG conception, design. OS, CD’E, LM, CG, ML and VV acquisition of data. OS and TC analysis of data. OS and JLG interpretation of data. AS and SS drafting the manuscript. GC, AM and MC revising the manuscript. JLG critically for important intellectual content. All authors read and approved the final manuscript.

## Supplementary Material

Additional file 1: Figure S1 Box plot showing differences in physical well being average score across urinary diversions. (1CUS, 2BK-IC, 3ONB-VIP). Click here for file

Additional file 2: Figure S2 Box plot showing differences in emotional well being average score across urinary diversions. (1CUS, 2BK-IC, 3ONB-VIP). Click here for file
