# Supplementary material for: Quality of life in women undergoing urinary diversion for bladder cancer: results of a multicenter study among long-term disease-free survivors
Source: Health Qual Life Outcomes. 2013 Mar 12;11:43. doi: 10.1186/1477-7525-11-43 (PMC3600042; doi:10.1186/1477-7525-11-43)
Supplement: Additional file 2: Figure S2 — Box plot showing differences in emotional well being average score across urinary diversions. (1CUS, 2BK-IC, 3ONB-VIP). [file 1477-7525-11-43-S2.pdf]

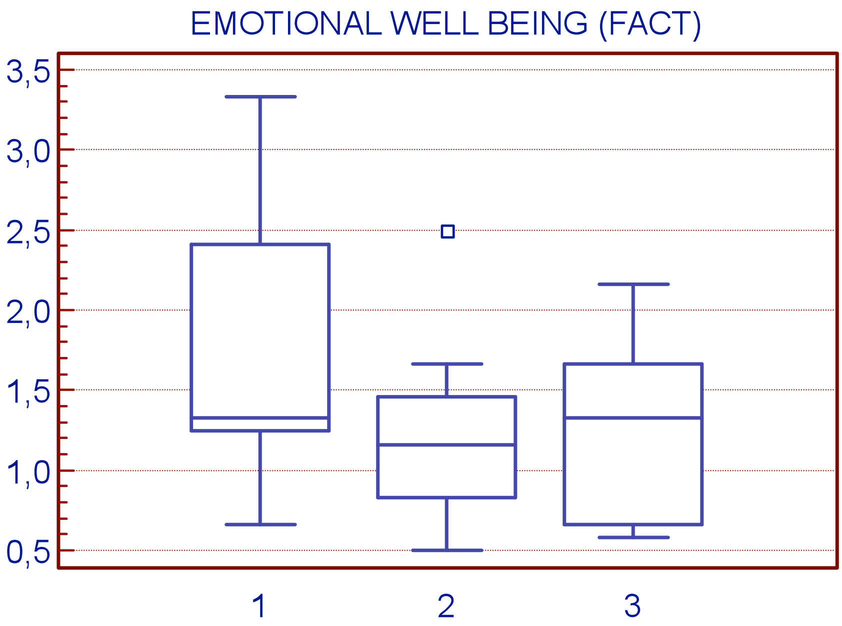

Figure 2: Box plot showing differences in emotional well being average score across urinary diversions. (1CUS, 2BK-IC, 3ONB-VIP)
